# Comparison of EMG, Video, and Actigraphy Signals for Detecting Motor Activity in REM Sleep Behavior Disorder

**DOI:** 10.3390/diagnostics16071067

**Published:** 2026-04-01

**Authors:** Kang Hyun Ryu, Giorgio Ricciardiello Mejia, Salonee Marwaha, Andreas Brink-Kjaer, Emmanuel H. During

**Affiliations:** 1 Department of Neurology, Icahn School of Medicine, New York, NY 10029, USA; kanghyun.ryu@mountsinai.org (K.H.R.); giorgio.ricciardiellomejia@mountsinai.org (G.R.M.); salonee.marwaha@icahn.mssm.edu (S.M.); 2 Department of Health and Technology, Technical University of Denmark, 2800 Kongens Lyngby, Denmark; andbri@dtu.dk; 3 Department of Medicine, Division of Pulmonary, Critical Care and Sleep Medicine, Icahn School of Medicine, New York, NY 10029, USA; 4 Department of Artificial Intelligence and Human Health, Icahn School of Medicine, New York, NY 10029, USA

**Keywords:** rapid-eye-movement sleep behavior disorder, sleep, actigraphy, electromyography, video-polysomnography

## Abstract

**Background:** Electromyography (EMG), video-polysomnography (vPSG), and wrist actigraphy are each used to develop diagnostic algorithms for rapid-eye-movement sleep behavior disorder (RBD). However, the extent to which they capture overlapping versus distinct motor phenomena remains unknown. We evaluated the respective contributions of actigraphy, EMG and vPSG to the measurement of REM sleep motor activity. **Methods:** Seventeen adults with RBD (Mount Sinai *n* = 9; Stanford *n* = 8) and eight control participants from an open Newcastle dataset underwent vPSG and concomitant wrist actigraphy. Flexor digitorum superficialis EMG activity and video-detected movements were manually scored in 3 s mini epochs. Actigraphy was quantified using an acceleration-magnitude-based activity count model. Statistical and agreement analyses were performed to assess the motor events captured by all three, any two, or by each modality independently during REM sleep. **Results:** In participants with RBD, actigraphy-derived movement load was significantly higher during REM sleep than during non-REM stages, a pattern not observed in control participants. REM movement load was also higher in RBD participants compared to controls, although this difference did not remain significant after correction for multiple comparisons. Across 12,941 3 s mini epochs, EMG, actigraphy, and video detected 1703, 1613, and 811 motor events, of which 413 were detected concurrently by all three modalities. Pairwise agreement was moderate and increased from EMG–actigraphy (κ = 0.27 ± 0.10) to actigraphy–video (κ = 0.41 ± 0.12) and EMG–video (κ = 0.45 ± 0.15). Of EMG-detected events, 49.0% were also detected by actigraphy; of actigraphy-detected events, 37.2% were detected by EMG and 34.9% by video. Actigraphy activity counts were highest for events detected by all three modalities and lowest for actigraphy-only events. **Conclusions:** Actigraphy-measured REM-related motor activity was elevated in RBD but not in controls. EMG, actigraphy, and video captured partially overlapping motor events in RBD patients, with actigraphy showing the highest sensitivity and manually scored video the lowest.

## 1. Introduction

Rapid-eye-movement sleep behavior disorder (RBD) is a parasomnia characterized by the loss of normal muscle atonia during REM sleep, resulting in motor disinhibition [1]. Motor manifestations in RBD vary widely in intensity, both across individuals and from night to night, ranging from subtle twitches to complex and occasionally violent behaviors. Prompt and accurate diagnosis of RBD is critical, as it is most often a prodromal manifestation of synucleinopathies, including Parkinson’s disease, dementia with Lewy bodies, and multiple system atrophy.

The diagnosis of RBD relies on overnight video-polysomnography (vPSG). This assessment includes electromyography (EMG) recordings from the submentalis (chin), flexor digitorum superficialis (upper extremities), and anterior tibialis muscles (lower extremities) to quantify muscle activity during REM sleep, along with synchronized infrared video recording, as outlined by International RBD Study Group guidelines [2]. EMG provides a direct window into the pathophysiologic hallmark of RBD—excessive muscle activity during REM sleep—which reflects well-characterized disruptions in REM sleep motor control circuits, including dysfunction within pontomedullary inhibitory circuits and altered cortical–brainstem interactions [3]. In contrast, video data are particularly valuable for evaluating RBD symptom severity [4,5,6].

Importantly, EMG activity does not always correspond to overt movements, as some electrical activity may not reach a movement threshold. Conversely, not all movements detected on video are captured by EMG, as they may involve muscle groups that are not monitored. Yet, movements in video may be obscured by bed coverings, and subtle twitches may fall below the detection threshold of infrared cameras. Recent studies have explored the potential of video analysis for differentiating RBD from other sleep disorders [7,8].

A third modality, wrist actigraphy, has been shown to capture excessive movements in RBD compared to other sleep disorders and healthy sleep [9,10,11]. More recent work has extended these approaches using automated and machine learning-based methods for RBD detection across home and ambulatory settings, with actigraphy-based classifications validated against concurrently acquired vPSG recordings [12,13,14,15,16]. Despite a growing body of evidence supporting the development of actigraphy-based classifiers—and the potential scalability of this approach as a screening tool—two critical aspects of wrist accelerometry in RBD remain insufficiently studied: (1) the extent to which movements detected in RBD are specific to REM sleep, as opposed to non-REM sleep or wakefulness; and (2) the degree to which wrist actigraphy captures overlapping versus distinct motor phenomena of REM sleep when compared with EMG–particularly of the upper extremities, and with movements observed on video.

## 2. Materials and Methods

### 2.1. Participants

Three research cohorts were used in this study:

a. RBD cases. 17 adult participants with RBD (*n* = 9) at Mount Sinai Sleep Research Center, (*n* = 8) at the Stanford Sleep Research Center, (*n* = 16) with idiopathic RBD, (*n* = 1) with antidepressant-induced RBD, underwent vPSG with concomitant wrist actigraphy between June 2021 and November 2025. All RBD diagnoses were confirmed by overnight vPSG based on ICSD-3 criteria [17]. Research participants signed an informed consent form, and the study was approved by the Institutional Review Boards of the Icahn School of Medicine at Mount Sinai. Participant demographics are summarized in Table 1 below.

b. Controls. The open research Newcastle dataset [18] (*n* = 8), in which participants also wore wrist actigraphy during concomitant vPSG, was used for this study.

The Mount Sinai RBD cohort served as the primary dataset for multimodal comparisons (EMG, actigraphy, and video), as it was the only cohort with fully synchronized recordings across all three modalities. The Stanford RBD cohort was combined with the Mount Sinai cohort exclusively for analyses of actigraphy-derived movement load across sleep stages (REM vs. non-REM (NREM)) to increase statistical power. Stanford data were acquired through an ongoing collaboration with the Stanford Sleep Center and collected under standardized protocols. The Newcastle control dataset was used solely for between-group comparisons of actigraphy-derived movement load (RBD vs. controls).

Accordingly, analyses of movement load across sleep stages (Result Section 3.1) included the combined RBD cohort (Mount Sinai + Stanford) and controls, whereas the multimodal overlap analyses (Result Section 3.2, Section 3.3 and Section 3.4) were restricted to the Mount Sinai cohort to ensure consistency in multimodal acquisition and scoring.

### 2.2. Video PSG

All RBD participants were connected to PSG equipment 2 h prior to their habitual bedtime, which was determined by participant interview. Data (diagnostic or on a therapeutic Continuous Positive Airway Pressure (CPAP) machine) was acquired following standard American Academy of Sleep Medicine (AASM) protocols using Compumedics E-series and Grael 2 systems (Compumedics Ltd., Melbourne, Australia). Signal acquisition included 19 channel electroencephalography placed according to the International 10–20 system or a subset of 6 channels (F3, F4, C3, C4, O1, and O2), left and right electro-oculography (EOG), all sampled at 256 or 512 Hz and referenced to a common patient and ground electrode, submental bipolar, bilateral flexor digitorum superficialis, and anterior tibialis bipolar EMG, respiration by a pressure transducer and PAP device interface, effort by rib/abdomen impedance plethysmography, single-channel ECG, and oxygen saturation by pulse oximetry (SpO2). Synchronized infrared video was recorded continuously throughout the night.

Sleep stages were scored by a sleep technician in 30 s epochs according to standard criteria for sleep and EEG arousals and confirmed by a board-certified sleep specialist. Total sleep time (TST) and percentage time spent in wake, REM sleep, non-REM stage 1 (N1), non-REM stage 2 (N2), and slow-wave sleep (SWS/N3) were determined.

A summary of the Mount Sinai, Stanford, and Newcastle vPSG data analyzed in this study is presented in Table 2, Tables S1 and S2. REM sleep periods shorter than 5 min were excluded from analysis, in line with International RBD Study Group guidelines [2]. Consequently, one REM period was excluded for Mount Sinai participant 6 (leaving three REM periods) and for control participants 1 and 2 (leaving two REM periods each).

### 2.3. Actigraphy

**Figure 1:**
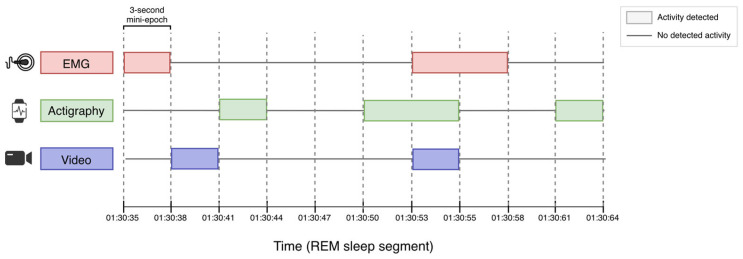
Study design and epoch-level synchronization. Synchronized bilateral flexor digitorum superficialis electromyography (EMG), wrist actigraphy, and infrared video were recorded during overnight video-polysomnography (vPSG) at the Mount Sinai Sleep Research Center. Motor activity detected by each modality was scored in contiguous 3 s mini-epochs during REM sleep, with timestamps aligned using a standardized brisk arm movement synchronization maneuver. Dashed vertical lines indicate mini-epoch boundaries. Colored blocks represent mini-epochs in which activity was detected by each modality (EMG: red; actigraphy: green; video: blue), while unshaded segments indicate no detected activity.

All RBD participants wore an AX6 triaxial accelerometer (Axivity Ltd., Newcastle, UK) on both wrists during their overnight vPSG, recorded at 50 Hz with a ±8 g dynamic range. Timestamps across EMG, infrared video, and actigraphy were synchronized by aligning the prominent movement artifacts generated by a standardized brisk arm maneuver performed at the start of the vPSG recording. An illustrative example of the study design is shown in Figure 1.

All control participants in the Newcastle dataset wore a GENEActive actigraph [18] (Activinsights Ltd., Kimbolton, UK). To ensure comparability across datasets, both devices were configured with the same dynamic range (±8 g), and gyroscope data were not used [19,20]. Raw acceleration signals were processed using harmonized pipelines, including resampling to a common frequency of 25 Hz prior to feature extraction.

Although preprocessing steps were aligned, the devices differ in native sampling frequency (85.7 Hz for GENEActive vs. 50 Hz for AX6) and resolution (12-bit vs. 16-bit, respectively). These hardware differences may influence sensitivity to low-amplitude movements and introduce subtle differences in movement quantification across devices.

### 2.4. Electromyographic Scoring

Bilateral flexor digitorum superficialis EMG activity during REM sleep was scored according to the AASM Manual for the Scoring of Sleep and Associated Events, Version 3 [21]. EMG signals were filtered using a 5 Hz high-pass filter and a 500 Hz low-pass filter. Within each REM sleep period, EMG activity was manually scored in 3 s mini-epochs using a binary system. Activity was defined as an amplitude at least twice the participant’s baseline atonia level for that REM sleep cycle and lasted between 0.1 and 5.0 s. The atonia level for each REM period was defined as the lowest EMG amplitude observed during that period, or if lower, during the preceding non-REM period.

### 2.5. Video Scoring

Arm movements during each REM sleep cycle were manually evaluated from the video recordings and scored using a binary system. Video scoring was performed blinded to the EMG and actigraphy scores to minimize bias. Any observable arm movement within each 3 s mini-epoch was marked as activity. Movements were not differentiated by amplitude; small twitches and large, full-body movements were treated equivalently and coded as 1 (activity).

### 2.6. Actigraphy Scoring: Activity Counts

Activity counts from the accelerometer data were calculated following the activity count model described in Brink-Kjaer et al. (2022) [12]. In brief, raw accelerometer data was calibrated such that the vector magnitude is equal to the gravitational acceleration (1 g) during periods of inactivity. Activity counts were calculated as the acceleration magnitude based on the Euclidean Norm Minus One formula:

$$Activity\;Count_{i}\;=\;\left|\sqrt{{x_{i}}^{2}+{y_{i}}^{2}+{z_{i}}^{2}\;}\;-\;1\right|$$

where $x_{i}$, $y_{i}$, and $z_{i}\;$ represent acceleration along the three orthogonal axes. The activity counts were then averaged in 1 s bins. To mitigate baseline drift, the resulting time series were forward–backward filtered using a high-pass infinite impulse response (IIR) Butterworth filter with a stopband attenuation of 80 dB at 0.1 Hz and passband ripple of 1 dB starting at 0.5 Hz. Activity counts less than 0.1 were rounded down to zero. A 3 s mini epoch was classified as active if the maximum activity count within the epoch exceeded zero.

Accelerometer data from control participants in the Newcastle dataset were processed using a modified version of the open-dataset processing code [18,22], and activity counts were calculated using the same algorithm described above.

### 2.7. Statistical and Agreement Analysis

Signal processing and statistical analyses were performed using MATLAB (R2024b, The MathWorks, Natick, MA, USA) and Python (version 3.11, Python Software Foundation, Wilmington, DE, USA). Differences in actigraphy-derived activity counts across sleep stages were first examined to evaluate whether detected movements were specific to REM sleep rather than non-REM sleep and wakefulness, and whether REM motor activity was elevated in participants with RBD relative to controls.

We quantified actigraphy-derived movement load, defined as the sum of left and right wrist activity counts per minute, for each sleep stage. Within-participant pairwise comparisons of movement load between sleep stages were conducted using Wilcoxon signed-rank tests, with Holm *p* value correction for multiple comparisons.

Because motor activity-event prevalence differed across participants and modalities, we prioritized agreement metrics that are more robust to marginal imbalance. Cross-modality agreement was quantified using Cohen’s κ (chance-corrected agreement) [23]. We also calculated directional conditional probabilities (e.g., P(Actigraphy = 1∣EMG = 1) and P(Video = 1|Actigraphy = 1)) between EMG, actigraphy, and video pairs to characterize their co-detection patterns.

Agreement and conditional probability analyses were performed at the level of 3 s mini-epochs. Multi-epoch motor events spanning consecutive epochs were treated as independent active epochs for these analyses. Left and right wrist detections were combined such that an epoch was classified as active if activity was detected in either wrist. Group-level 95% confidence intervals for Cohen’s κ and conditional probabilities were estimated across participants using nonparametric bootstrap resampling.

In addition, modality-detected motor activity rates and ratios were compared to quantify the proportion of activity captured by each modality. All metrics were computed per participant and then summarized across participants.

Finally, to assess whether actigraphy movement magnitude varied with multimodal consensus, we compared accelerometer-derived activity counts across mutually exclusive multimodal agreement categories (actigraphy alone; actigraphy and video; EMG and actigraphy; and all three modalities). Specifically, REM mini-epochs were stratified by overlapping detection condition, and for each category we computed the mean actigraphy activity count across epochs. These values were then compared across conditions to assess how actigraphy-detected movement intensity changed as a function of modality co-detection.

## 3. Results

### 3.1. Motor Activity During REM vs. NREM

**Figure 2:**
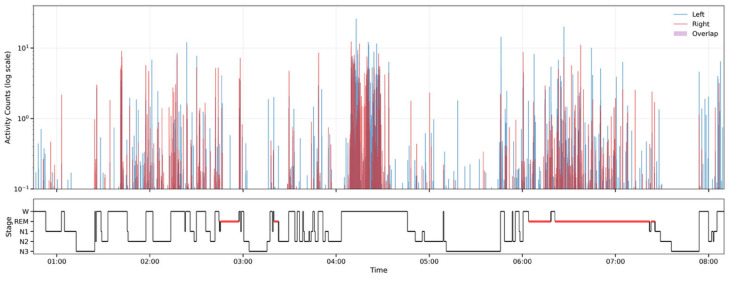
Sample bilateral wrist actigraphy aligned to sleep stages in an RBD participant (Mount Sinai participant 2). The upper panel shows left (blue) and right (red) wrist activity counts (log scale) across the overnight recording; purple shading marks epochs with concurrent activity in both wrists (overlap). The lower panel shows the corresponding hypnogram (W, N1, N2, N3, REM), with REM periods highlighted in red. Plots for the remaining Mount Sinai participants are provided in Supplementary Figures S1 and S2.

Bilateral wrist actigraphy activity counts demonstrated sleep–wake state-dependent variability in participants with RBD (Figure 2, Figures S1 and S2). Activity was highest in mean amplitude (0.233 ± 0.113) and frequency (14.0 ± 2.77) during wake and was generally lower and sparser across NREM sleep (amplitude 0.027 ± 0.013; frequency 2.88 ± 1.40). REM epochs exhibited intermittent clusters of elevated activity relative to NREM.

**Figure 3:**
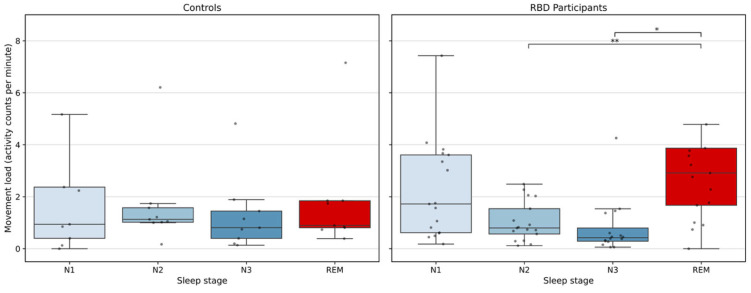
Movement load across sleep stages in control (**left**) and RBD participants (**right**). Movement load, defined as the sum of left and right wrist actigraphy activity counts per minute, is shown for N1 (light blue), N2 (blue), N3 (dark blue), and REM (red) sleep. Each dot represents a single participant, and boxplots summarize the distribution across participants. Pairwise comparisons were conducted using Wilcoxon signed-rank tests with Holm correction for multiple comparisons; asterisks denote statistical significance (* *p* < 0.05, ** *p* < 0.01). For RBD patients, movement load during REM sleep was significantly greater than during N2 (adjusted *p* = 0.003) and N3 sleep (adjusted *p* = 0.019). Note. A small number of higher values were truncated in the display to improve scale and readability; full distributions are shown in Supplementary Figure S5.

In control participants, actigraphy movement load was low across all sleep stages, with median values of 0.94 [IQR 1.97] in N1, 1.13 [IQR 0.56] in N2, 0.81 [IQR 1.05] in N3, and 0.90 [IQR 1.04] in REM sleep (see Figure 3 and Figure S5). No significant differences were observed between the NREM and REM stages. In contrast, in RBD participants, significant increase was observed in REM relative to NREM sleep. The movement load was highest during REM sleep (median 2.91 [IQR 2.20]), followed by N1 (median 1.72 [IQR 2.99]), N2 (median 0.80 [IQR 0.97]), and N3 sleep (median 0.42 [IQR 0.51]) with statistically significant differences in REM sleep compared to N2 (adjusted *p* = 0.003) and N3 sleep (adjusted *p* = 0.019). Movement load during REM sleep was higher in RBD participants than in controls (Mann–Whitney U test, uncorrected *p* = 0.046), with a large effect size (Cliff’s δ = 0.49). However, this effect did not remain significant after false discovery rate correction across sleep stages (adjusted *p* = 0.185).

### 3.2. Comparison of Motor Activity Detected by EMG, Actigraphy and Video

**Figure 4:**
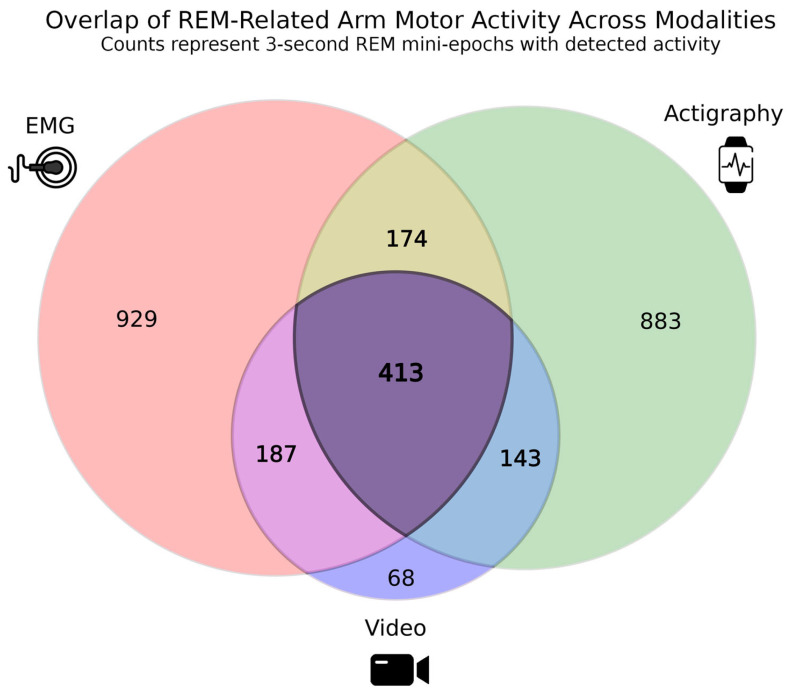
Overlap of REM-related motor activity detected by EMG, actigraphy, and video. This diagram illustrates, among the 12,941 three-second REM mini-epochs recorded across all participants, the number of epochs in which motor activity was detected by EMG (red), wrist actigraphy (green), and infrared video (blue) on the left, right, or both sides of the arm. Each region represents the number of mini-epochs with activity detected uniquely by one modality or concurrently by two or all three modalities. A total of 929 epochs were detected by EMG only, 883 by actigraphy only, and 68 by video only, while 413 epochs were detected by all three modalities.

Across 12,941 3 s REM mini-epochs recorded across participants, 2797 contained motor events detected on the left, right, or both sides—captured by one, two, or all modalities. Of these, EMG, wrist actigraphy, and video detected 1703, 1613, and 811 motor events, respectively (Figure 4). Of these motor events, 413 events were detected simultaneously by all three modalities. A total of 174 events were detected by both EMG and actigraphy, 187 events were detected by EMG and video, and 143 events were detected by actigraphy and video. Modality-specific detections were also observed, with 929 events detected by EMG alone, 883 by actigraphy alone, and 68 by video alone.

Agreement between modalities during REM sleep was assessed using Cohen’s κ (Table 3; Figure S3). Overall, pairwise agreement was moderate, with mean κ values increasing from EMG–actigraphy (κ = 0.27, 95% CI [0.21, 0.33]) to actigraphy–video (κ = 0.41, 95% CI [0.34, 0.49]) and EMG–video (κ = 0.45, 95% CI [0.36, 0.54]). Consistent with these results, odds ratio analyses (log scale) showed the strongest association between EMG–video (pooled OR ≈ 49, 95% CI [29.8, 80.3]), followed by actigraphy–video (pooled OR ≈ 33, 95% CI [16.6, 66.9]) (Figure S4). EMG–actigraphy exhibited the weakest, but still robust and positive association (pooled OR ≈ 10.6, 95% CI [6.26, 17.9]).

**Figure 5:**
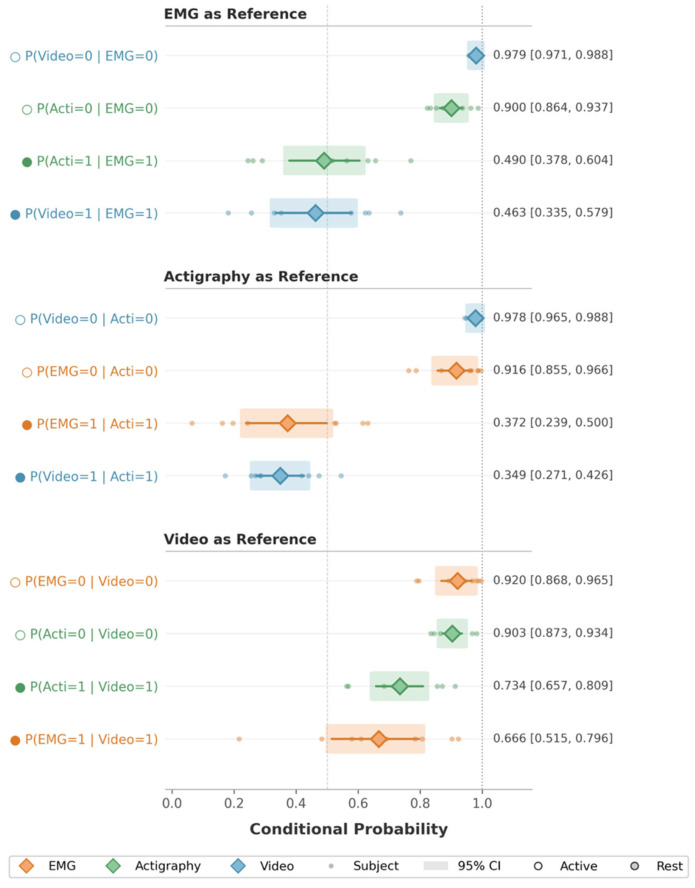
Bidirectional conditional probabilities of REM-related motor activity detection across EMG, actigraphy, and video. For each modality pair, directional conditional probabilities (e.g., P(A = 1|B = 1) and P(B = 1|A = 1)) were computed to quantify cross-modal co-detection during REM sleep. Diamond markers indicate mean conditional probabilities across participants, with error bars representing 95% confidence intervals.

We quantified relationships between modalities using directional conditional probabilities to characterize cross-modal concordance (Figure 5, Tables S3–S5). Across participants, actigraphy and video showed asymmetric patterns of agreement with EMG. The probability that actigraphy detected motor activity given concurrent EMG activity (P(Acti = 1|EMG = 1)) was modest (mean 0.49, 95% CI [0.38, 0.60]), indicating that actigraphy captured approximately half of EMG-associated motor events. Similarly, the probability that video detected activity given EMG activity (P(Video = 1|EMG = 1)) was slightly lower (0.46, 95% CI [0.34, 0.58]).

In contrast, conditional probabilities in the reverse direction were substantially lower for actigraphy than for video. The probability that EMG activity was present when actigraphy detected movement (P(EMG = 1|Acti = 1)) was 0.37, 95% CI [0.24, 0.50], whereas video detections were more frequently accompanied by EMG activity (P(EMG = 1|Video = 1) = 0.67, 95% CI [0.52, 0.80]).

The probability that actigraphy detected motor activity when video detected activity was relatively high (P(Acti = 1|Video = 1) = 0.73, 95% CI [0.66, 0.81]), indicating that most visually apparent movements were also captured by actigraphy. In contrast, the probability that video detected activity when actigraphy detected movement was substantially lower (P(Video = 1|Acti = 1) = 0.35, 95% CI [0.27, 0.43]), suggesting that a large proportion of actigraphy-detected movements were not visually apparent on infrared video.

Across all modality pairs, probabilities of concordant absence (e.g., P(Acti = 0|EMG = 0), P(Video = 0|Acti = 0)) were uniformly high (above 0.90).

### 3.3. REM Motor Activity Ratio and Rate Measured Across Modalities

To compare the overall burden of REM-related motor activity detected by each modality, we measured an “activity ratio” and an “activity rate” (Table 4). Activity ratio is defined as the percentage of REM sleep mini-epochs that contain any detected arm activity. Activity rate is defined as the number of distinct motor events per hour of REM sleep. A motor event is counted once regardless of its duration: events may be brief (lasting <3 s and confined to a single mini-epoch) or prolonged (extending across multiple consecutive 3 s mini-epochs) but are considered a single event from onset to offset.

Across participants, actigraphy detected the highest overall motor activity burden by both metrics, with a mean activity ratio of 13.7 ± 6.6% and a rate of 70.0 ± 31.7 per hour. EMG detected activity during a similar proportion of REM epochs (12.0 ± 10.3%) with a comparable rate (66.8 ± 47.1 events per hour), whereas video detected activity during a smaller proportion of epochs (6.6 ± 4.4%) and at substantially lower rates (38.8 ± 22.4 events per hour). Activity rates showed high inter-individual variability.

### 3.4. Actigraphy Activity Counts Across Overlapping Detection Conditions

**Figure 6:**
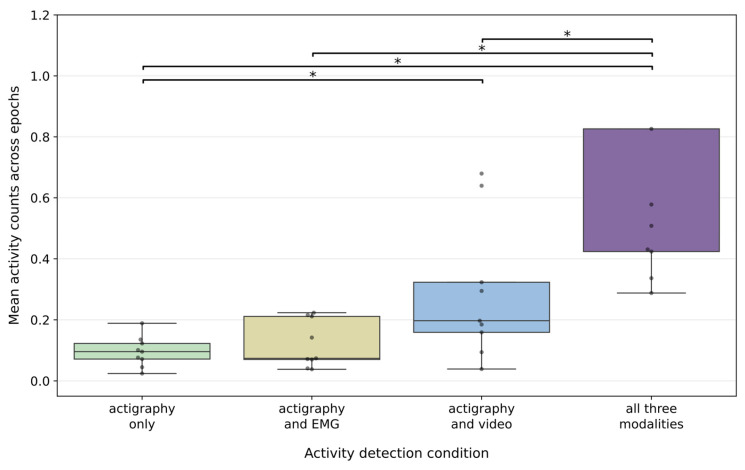
Distribution of mean actigraphy activity counts during REM sleep epochs stratified by multimodal detection condition: epochs detected by all three modalities (EMG, actigraphy, and video), by actigraphy and EMG, by actigraphy and video, or by actigraphy alone. Each dot represents a single Mount Sinai RBD participant. Actigraphy activity counts were highest for epochs detected by all three modalities and lowest for actigraphy-only epochs. Pairwise comparisons were performed using Wilcoxon signed-rank test with Holm correction; asterisks denote statistically significant differences (* *p* < 0.05). Note. A small number of higher values were truncated in the display to improve scale and readability; full distributions are shown in Supplementary Figure S6.

Actigraphy activity counts were highest for epochs in which motor activity was detected by all three modalities (median 0.508 [IQR 0.402]) (Figure 6 and Figure S6). Intermediate activity levels were observed for epochs detected by actigraphy–video pairs (median 0.197 [IQR 0.164]) and EMG–actigraphy pairs (median 0.0740 [IQR 0.141]). The lowest activity counts were observed for actigraphy-only epochs (median = 0.0958 [IQR 0.0511]).

Wilcoxon signed-rank test with Holm correction for multiple comparisons demonstrated that actigraphy activity counts for all three detections condition were significantly greater than those for actigraphy–video, EMG–actigraphy, and actigraphy-only epochs, while differences between EMG–actigraphy and actigraphy–video conditions were not statistically significant. Together, these findings indicate that higher-amplitude actigraphy signals are more likely to correspond to motor events detected by multiple modalities, whereas lower-amplitude actigraphy signals are more frequently detected in isolation.

## 4. Discussion

In this study, we directly compared EMG, wrist actigraphy, and infrared video within synchronized REM sleep epochs to characterize how each modality captures motor activity in patients with RBD. Actigraphy-derived movement load was significantly higher during REM sleep compared to NREM stages, a pattern that was not observed in control participants. This REM-specific elevation aligns with the loss of REM atonia characteristic of RBD and demonstrates that actigraphy can capture disease-relevant motor activity during REM sleep.

Overall, agreement between video and the other two modalities—EMG (κ = 0.45 ± 0.15) and actigraphy (κ = 0.41 ± 0.12)—was highest, consistent with the notion that movements visible on video tend to be larger motor events that are more likely to involve both muscle activation and limb displacement. Conversely, disagreement between video and the other modalities may reflect limitations of visual detection, as some movements were obscured by bedding or body position, or occurred with minimal visible displacement despite underlying muscle activation or acceleration.

Our findings show moderate agreement between actigraphy and EMG-defined motor activity, with actigraphy detecting approximately half of the epochs containing EMG activity (P(Acti = 1|EMG = 1) = 0.49). Probabilities of concordant absence were uniformly high (above 0.90) across all modalities, reflecting strong agreement during periods without detectable motor activity, likely driven by the predominance of inactive REM epochs. Actigraphy also detected motor activity in a substantial proportion of epochs without concurrent EMG activity (63% of actigraphy-detected activity), likely reflecting sensitivity to movement signals that may not be captured by the recorded EMG channels. In addition, actigraphy yielded the highest overall motor activity burden, as reflected by greater activity ratios (13.7 ± 6.6%) and event rates (70.0 ± 31.7 events per hour) compared with EMG and video. Together, these findings suggest that actigraphy captures a broader range of movement-related signals, including both multimodally concordant events and movements detected in isolation.

Modality-specific detections were observed across all three modalities and likely reflect differences in the physiological signals captured by each method. EMG-only events, which accounted for a substantial proportion of detections (929 epochs), may represent tonic flexor digitorum superficialis activation that do not result in sufficient wrist acceleration or visible displacement. In contrast, actigraphy-only detections may reflect movements involving proximal or axial musculature, or whole-body motion, that are not captured by forearm EMG recordings. Video-only events, although less frequent, may represent visually apparent movements involving muscle groups not captured by the recorded EMG channels or movements with low-amplitude or sustained acceleration that do not exceed actigraphy detection thresholds. These findings highlight that each modality captures complementary aspects of motor activity.

Further analysis showed that actigraphy activity counts were the highest (median 0.508 [IQR 0.402]) for epochs in which motor activity was detected concurrently by all three modalities and the lowest (median 0.0958 [IQR 0.0511]) for actigraphy-only epochs, indicating that activity with higher activity counts is more likely to reflect larger, overt, multimodally detectable movements. In contrast, lower activity counts were frequently detected in isolation, suggesting that actigraphy signal magnitude may serve as a useful proxy for movement salience. These findings have practical implications for actigraphy-based RBD detection algorithms, including the potential utility of incorporating amplitude-based thresholds to improve specificity for clinically meaningful motor events.

Several limitations should be considered when interpreting these results. First, the sample (Mount Sinai cohort) size was small and included only male participants, which may limit generalizability. Second, the control group wore a different type of device, which may introduce calibration or sensitivity differences that complicate comparisons with the RBD participants. Additionally, differences in participant populations and study protocols across institutions may introduce further variability. Third, motor activity was scored using binary criteria within 3 s mini-epochs, which do not capture individual movements’ duration or complexity. The use of mini-epochs may also have influenced agreement estimates, as longer movements may be fragmented across adjacent bins and small temporal misalignments (e.g., a 1 s offset) between modalities may lead to apparent disagreement within such short epochs. Incorporating event-level analyses, including movement duration and complexity, may provide a more detailed characterization of cross-modal agreement. vPSG scoring was performed manually by a single rater, which may introduce human error and limits assessment of inter-rater reliability. Future studies could incorporate automated analysis pipelines to reduce observer bias and improve reproducibility in vPSG scoring, as emerging work is actively exploring and validating such approaches [7,8,15,24,25]. In addition, all visible arm movements were treated equivalently without differentiation by amplitude or kinematic features, limiting more detailed characterization of video-detected activity. Finally, analyses of EMG recordings were restricted to the flexor digitorum superficialis to enable direct one-to-one comparisons with wrist actigraphy, whereas clinical vPSG assessments of RBD typically incorporate both chin and limb EMG channels for diagnosis.

Despite these limitations, our work provides a quantitative comparison of EMG, actigraphy, and video within the same REM sleep periods, extending prior RBD diagnostic and actigraphy studies to the level of motor-event concordance across modalities. These findings should be interpreted as preliminary evidence, offering new framework for quantitatively understanding the complementary strengths of EMG, video, and actigraphy. This framework may help inform the potential role of actigraphy in scalable RBD screening or longitudinal monitoring outside of the laboratory. Further work is needed to validate these findings in larger and more diverse cohorts. Additional studies could compare EMG with actigraphy across various body placements (wrist, trunk, ankles) and 3D time-of-flight video, which is more sensitive than conventional infrared video systems [8].

## Figures and Tables

**Table 1:** Participant demographics and clinical characteristics. Values are reported as mean ± standard deviation or number of participants (*n*), as indicated. iRBD = idiopathic REM sleep behavior disorder; OSA = obstructive sleep apnea, as defined by ≥ 5 apneas and/or hypopneas resulting in ≥4% oxygen desaturations per hour of sleep; RLS = restless legs syndrome; PLM = periodic limb movements, as defined by a PLM index ≥ 15 per hour of sleep.

	Mount Sinai RBD Participants (*n* = 9)	Stanford RBD Participants (*n* = 8)	Control Participants (*n* = 9)
Age (years)	57.5 ± 14.3	65.6 ± 8.23	49.4 ± 12.8
Sex (male, *n*)	9	7	7
BMI (kg/m^2^)	26.3 ± 2.86	28.5 ± 5.95	N/A
iRBD (*n*)	8	8	0
antidepressant-induced RBD (*n*)	1	0	0
Years from symptom onset	5 ± 6.09 *	N/A	N/A
Melatonin (*n*)	2	4	N/A
Clonazepam (*n*)	0	3	N/A
OSA (*n*)	3	0	1
RLS (*n*)	2	0	0
PLM (*n*)	1	2	N/A

* *n* = 8. N/A indicates variables that were not collected or not available for the given cohort.

**Table 2:** Sleep architecture characteristics for each RBD participant in the Mount Sinai cohort. Total sleep time, total REM sleep duration (with percentage of total sleep time), and number of REM sleep periods during the recorded overnight video-polysomnography (vPSG) are shown for each participant.

Mount Sinai RBD Participant	Total Sleep Time (Minutes)	Total REM Duration (Minutes)	REM Sleep Periods (Counts)
1	273	51.55 (18.9%)	3
2	326	93.5 (28.7%)	2
3	312	42.55 (13.6%)	3
4	487.5	83.55 (17.1%)	3
5	413	97.85 (23.7%)	2
6	410.5	45 (11.0%)	3
7	404.5	83.5 (20.6%)	4
8	521.5	111.55 (21.4%)	4
9	392	38 (9.69%)	1

**Table 3:** Agreement between EMG, actigraphy, and video detections during REM sleep. Cohen’s κ values are shown for pairwise comparisons between EMG, actigraphy, and video for each participant. Mean, standard deviation, and 95% confidence intervals across participants are reported.

Mount Sinai RBD Participant	Cohen’s κ		
	EMG vs. Acti	EMG vs. Video	Acti vs. Video
1	0.25	0.58	0.34
2	0.08	0.28	0.21
3	0.40	0.41	0.34
4	0.34	0.27	0.60
5	0.27	0.32	0.48
6	0.39	0.59	0.54
7	0.24	0.58	0.39
8	0.19	0.39	0.43
9	0.27	0.63	0.36
Mean [95% CI]	0.27 [0.21, 0.33]	0.45 [0.36, 0.54]	0.41 [0.34, 0.49]
Std Dev	0.10	0.14	0.12

**Table 4:** Bilateral REM-related motor activity burden across EMG, actigraphy, and video during REM sleep. The proportion of REM sleep epochs containing motor activity on either left or right side (ratio, %) and the corresponding activity rate (events per hour) for each modality was computed for each participant. Mean, standard deviation, and 95% confidence intervals across participants are reported.

Mount Sinai RBD Participant	Activity Ratio (%)			Activity Rate (Events/Hour)		
	EMG	Acti	Video	EMG	Acti	Video
1	5.04	19.79	5.53	32.59	72.16	32.59
2	2.14	15.56	4.71	22.46	106.52	46.20
3	8.34	12.22	4.82	57.81	88.84	29.61
4	8.26	3.41	1.86	60.32	26.57	13.64
5	26.37	12.47	7.31	133.67	74.20	42.31
6	21.56	21.00	15.89	114.67	101.33	78.67
7	1.14	4.43	1.74	8.62	17.25	9.34
8	27.43	12.82	10.04	128.01	52.71	65.08
9	8.03	21.58	7.24	42.63	90.00	31.58
Mean [95% CI]	12.03 [5.99, 18.60]	13.70 [9.53, 17.77]	6.57 [4.03, 9.56]	66.76 [38.97, 96.72]	69.95 [48.93, 88.28]	38.78 [25.25, 53.33]
Std Dev	10.26	6.63	4.37	47.09	31.74	22.42

## Data Availability

Control data used in this study is openly available in Zenodo at 10.5281/zenodo.1160410. RBD participant data is available upon reasonable request from the authors due to participant privacy and confidentiality restrictions.

## Supplementary Materials

The following supporting information can be downloaded at https://www.mdpi.com/article/10.3390/diagnostics16071067/s1. Table S1: Sleep architecture characteristics for each RBD participant in the Stanford cohort. Table S2: Sleep architecture characteristics for each control participant in the Newcastle open dataset. Table S3: Conditional probabilities of motor activity detection between EMG and actigraphy for each Mount Sinai RBD participant. Table S4: Conditional probabilities of motor activity detection between EMG and video for each Mount Sinai RBD participant. Table S5: Conditional probabilities of motor activity detection between actigraphy and video for each Mount Sinai RBD participant. Figure S1: Bilateral wrist actigraphy aligned to sleep stages in Mount Sinai RBD participants (1, 3, 4, 5). Figure S2: Bilateral wrist actigraphy aligned to sleep stages in Mount Sinai RBD participants (6–9). Figure S3: Agreement between EMG, actigraphy, and video detections during REM sleep. Figure S4: Pairwise association between EMG, actigraphy, and video detections during REM sleep. Figure S5: Full distributions of actigraphy-derived movement load across sleep stages in control (left) and RBD participants (right). Figure S6: Full distributions of mean actigraphy activity counts during REM sleep epochs stratified by multimodal detection condition.
