# Supplementary material for: Comparison of EMG, Video, and Actigraphy Signals for Detecting Motor Activity in REM Sleep Behavior Disorder
Source: Diagnostics (Basel). 2026 Apr 1;16(7):1067. doi: 10.3390/diagnostics16071067 (PMC13073419; doi:10.3390/diagnostics16071067)
Supplement: Supplementary file 1 [file diagnostics-16-01067-s001.zip › diagnostics-4181632-supplementary.pdf]

Supplementary Information

Stanford RBD Participant	Total sleep time (minutes)	Total REM duration (minutes)	REM sleep periods (number)
1	415	121 (29.2%)	3
2	361.5	66 (18.3%)	4
3	400.5	52.5 (13.1%)	4
4	502.5	206.5 (41.1%)	3
5	408	140 (34.3%)	4
6	418.5	76.5 (18.3%)	2
7	210	47 (22.4%)	3
8	484	53.5 (11.1%)	3

Table S1. Sleep architecture characteristics for each RBD participant in the Stanford cohort. Total sleep time, total REM sleep duration (with percentage of total sleep time), and number of REM sleep periods during the recorded overnight video-polysomnography (vPSG) are shown for each participant.

Newcastle Control Participant	Total sleep time (minutes)	Total REM duration (minutes)	REM sleep periods (number)
1	427.5	61 (14.3%)	2
2	507.5	21.5 (4.24%)	2
3	316.5	45 (14.2%)	3
4	563.5	143.5 (25.5%)	4
5	464.5	75.5 (16.3%)	3
6	248.5	19.5 (7.85%)	1
7	483	113.5 (23.5%)	3
8	490.5	158.5 (32.3%)	4
9	445	88 (19.8%)	4

Table S2. Sleep architecture characteristics for each control participant in the Newcastle open dataset. Total sleep time, total REM sleep duration (with percentage of total sleep time), and number of REM sleep periods during the recorded overnight video-polysomnography (vPSG) are shown for each participant.

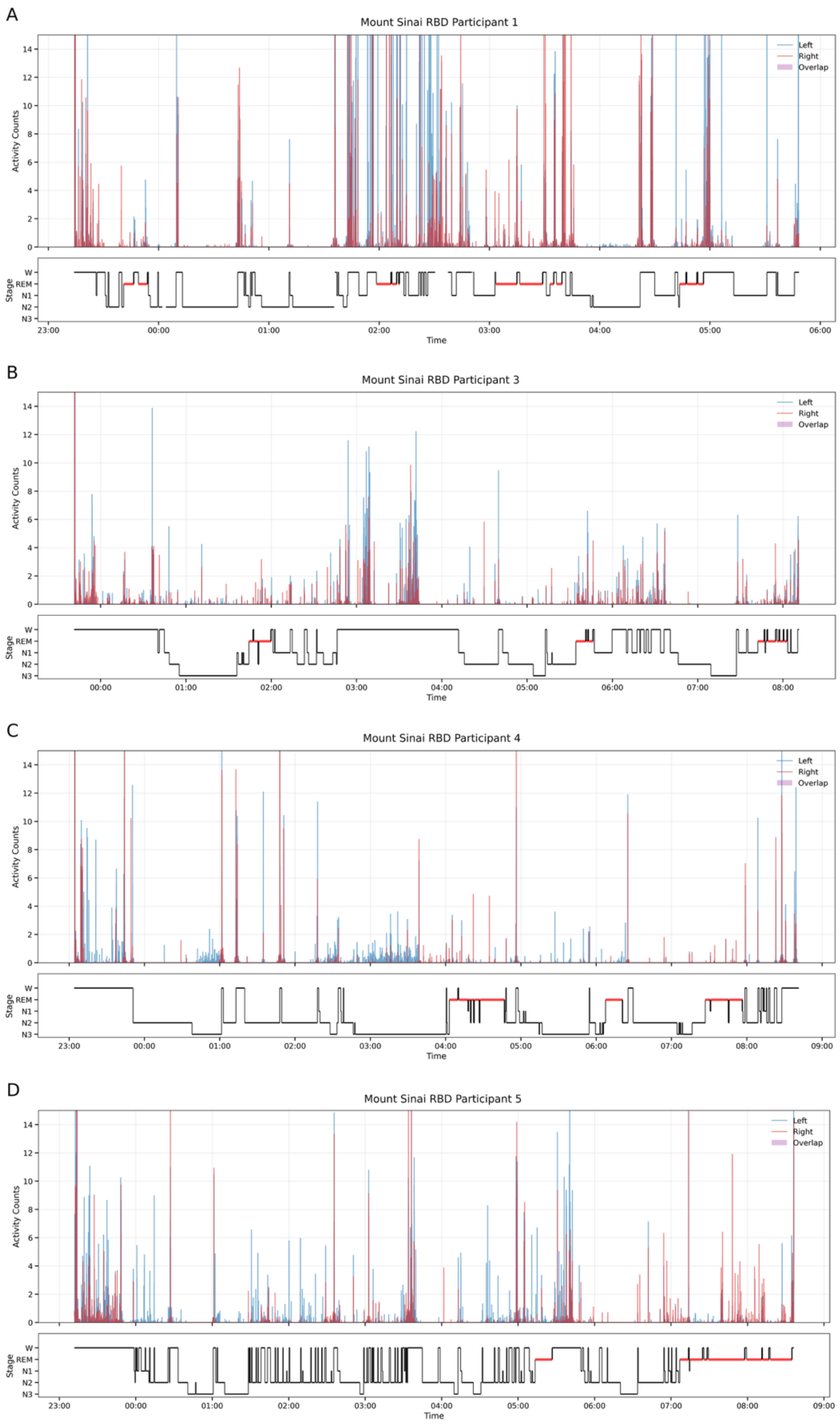

Figure S1. Bilateral wrist actigraphy aligned to sleep stages in Mount Sinai RBD participants (1, 3, 4, 5). Panels A-D correspond to participants 1, 3, 4, and 5, respectively. The upper panel shows left- (blue) and right-wrist (red) activity counts across the overnight recording; purple shading marks epochs with concurrent activity in both wrists (overlap). The lower panel shows the corresponding hypnogram (W, N1, N2, N3, REM), with REM periods highlighted in red. *Note.* The y-axis is truncated at 15 to improve readability.

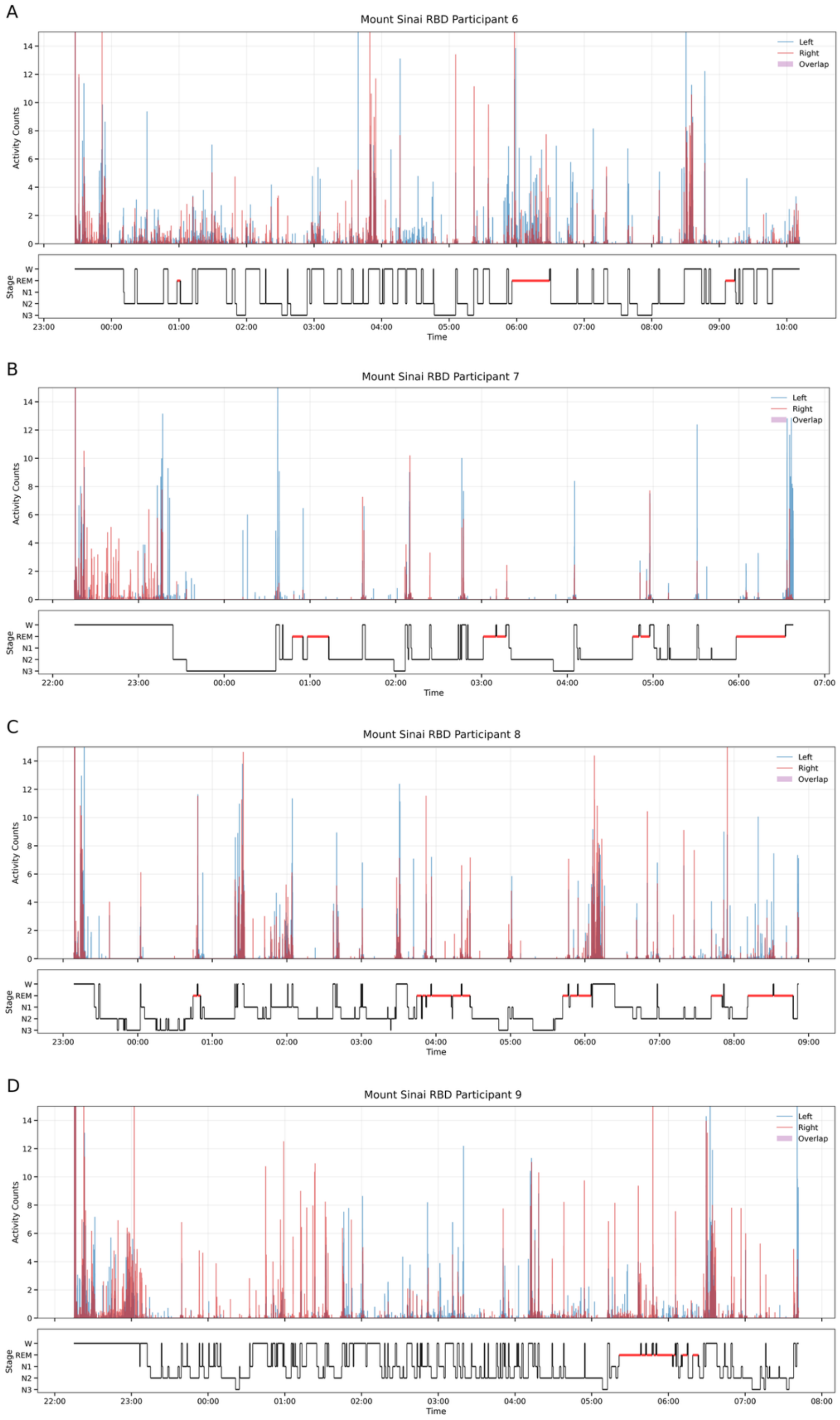

Figure S2. Bilateral wrist actigraphy aligned to sleep stages in Mount Sinai RBD participants (6-9). Panels A-D correspond to participants 6, 7, 8, and 9, respectively. The upper panel shows left- (blue) and right-wrist (red) activity counts across the overnight recording; purple shading marks epochs with concurrent activity in both wrists (overlap). The lower panel shows the corresponding hypnogram (W, N1, N2, N3, REM), with REM periods highlighted in red. *Note.* The y-axis is truncated at 15 to improve readability.

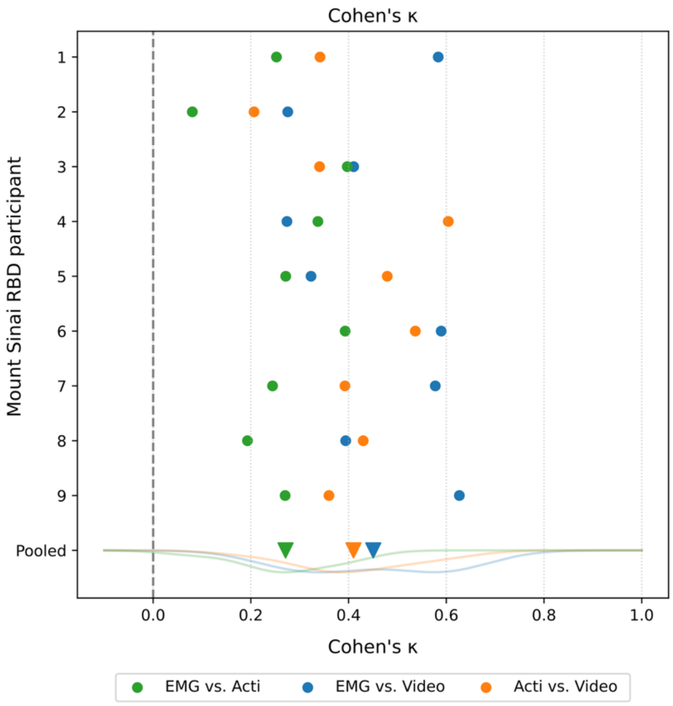

Figure S3. Agreement between EMG, actigraphy, and video detections during REM sleep. Cohen's κ is shown for pairwise comparisons between EMG and actigraphy (green), EMG and video (blue), and actigraphy and video (orange) for each participant. The downward triangles denote the pooled mean κ across participants for each comparison.

Mount Sinai RBD Participant	P(Acti=1 | EMG=1)	P(Acti=0 | EMG=0)	P(EMG=1 | Acti=1)	P(EMG=0 | Acti=0)
1	0.769	0.832	0.196	0.985
2	0.475	0.851	0.065	0.987
3	0.563	0.918	0.385	0.959
4	0.261	0.986	0.632	0.937
5	0.291	0.935	0.615	0.786
6	0.515	0.874	0.529	0.868
7	0.632	0.962	0.162	0.996
8	0.245	0.916	0.524	0.762
9	0.656	0.823	0.244	0.965
Mean [95% CI]	0.490 [0.378, 0.604]	0.900 [0.864, 0.937]	0.372 [0.239, 0.500]	0.916 [0.855, 0.966]
Std Dev	0.188	0.058	0.212	0.089

Table S3. Conditional probabilities of motor activity detection between EMG and actigraphy for each Mount Sinai RBD participant. Directional conditional probabilities (P(Acti=1 | EMG=1), P(Acti=0 | EMG=0), P(EMG=1 | Acti=1), and P(EMG=0 | Acti=0)) are shown for each participant. Values were computed at the 3-second REM mini-epoch level, with group-level mean (95% confidence interval) and standard deviation summarized across participants.

Mount Sinai RBD Participant	P(Video=1 | EMG=1)	P(Video=0 | EMG=0)	P(EMG=1|Video=1)	P(EMG=0 | Video=0)
1	0.635	0.975	0.579	0.980
2	0.475	0.962	0.216	0.988
3	0.352	0.979	0.610	0.943
4	0.181	0.996	0.806	0.931
5	0.256	0.992	0.923	0.788
6	0.577	0.956	0.783	0.892
7	0.737	0.991	0.483	0.997
8	0.330	0.986	0.902	0.796
9	0.623	0.976	0.691	0.967
Mean [95% CI]	0.463 [0.335, 0.579]	0.979 [0.971, 0.988]	0.666 [0.515, 0.796]	0.920 [0.868, 0.965]
Std Dev	0.192	0.014	0.224	0.080

Table S4. Conditional probabilities of motor activity detection between EMG and video for each Mount Sinai RBD participant. Directional conditional probabilities (P(Video=1 | EMG=1), P(Video=0 | EMG=0), P(EMG=1 | Video=1), and P(EMG=0 | Video=0)) are shown for each participant. Values were computed at the 3-second REM mini-epoch level, with group-level mean (95% confidence interval) and standard deviation summarized across participants.

Mount Sinai RBD Participant	P(Acti=1 | Video=1)	P(Acti=0 | Video=0)	P(Video=1 | Acti=1)	P(Video=0 | Acti=0)
1	0.912	0.844	0.255	0.994
2	0.568	0.865	0.172	0.976
3	0.683	0.906	0.269	0.983
4	0.871	0.982	0.474	0.998
5	0.713	0.922	0.418	0.976
6	0.720	0.886	0.545	0.944
7	0.724	0.968	0.284	0.995
8	0.563	0.920	0.441	0.950
9	0.855	0.834	0.287	0.987
Mean [95% CI]	0.734 [0.657, 0.809]	0.903 [0.873, 0.934]	0.349 [0.271, 0.426]	0.978 [0.965, 0.988]
Std Dev	0.125	0.051	0.123	0.019

Table S5. Conditional probabilities of motor activity detection between actigraphy and video for each Mount Sinai RBD participant. Directional conditional probabilities (P(Acti=1 | Video=1), P(Acti=0 | Video=0), P(Video=1 | Acti=1), and P(Video=0 | Acti=0)) are shown for each participant. Values were computed at the 3-second REM mini-epoch level, with group-level mean (95% confidence interval) and standard deviation summarized across participants.

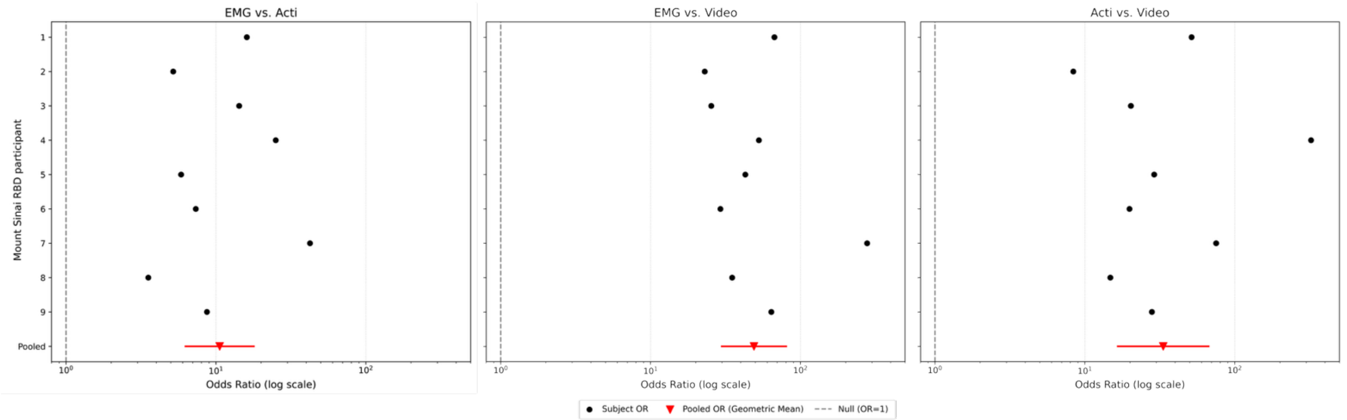

Figure S4. Pairwise association between EMG, actigraphy, and video detections during REM sleep. Odds ratios (ORs) are shown on a log scale for each Mount Sinai RBD participant (black dots) for EMG vs. actigraphy (left), EMG vs. video (middle), and actigraphy vs. video (right). The pooled OR across participants is shown in red (downward triangle) with its confidence interval (red horizontal line). The dashed vertical line indicates the null value (OR = 1).

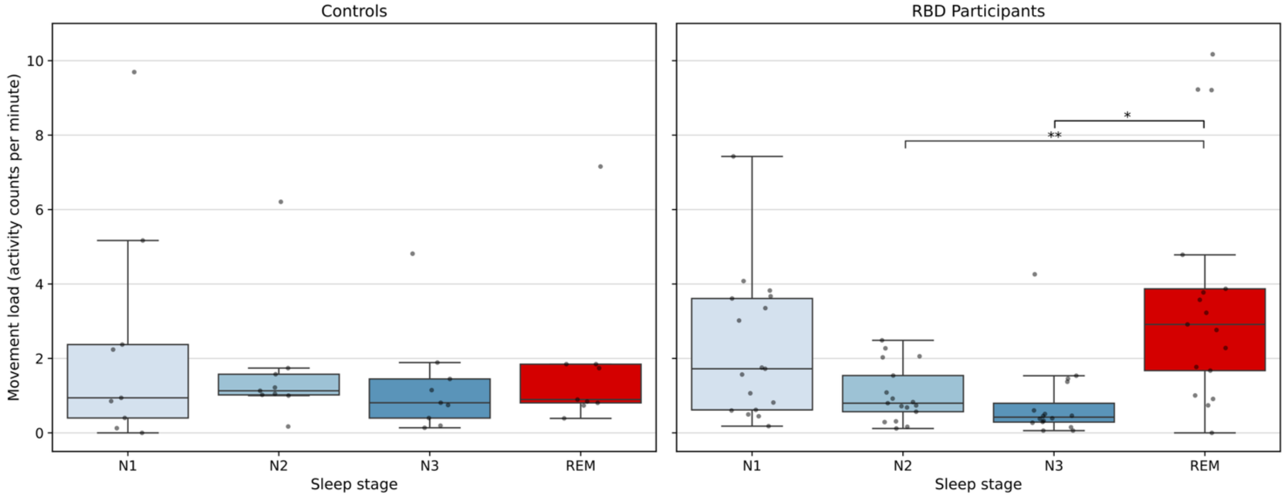

Figure S5. Full distributions of actigraphy-derived movement load across sleep stages in control (left) and RBD participants (right). Movement load, defined as the sum of left and right wrist actigraphy activity counts per minute, is shown for N1, N2, N3, and REM (R) sleep. Each dot represents a single participant, and boxplots summarize the distribution across participants. Pairwise comparisons were conducted using Wilcoxon signed-rank tests with Holm correction for multiple comparisons. For RBD participants, movement load during REM sleep was greater than during N2 and N3 sleep. This figure displays the complete (untruncated) distribution of values corresponding to Figure 3.

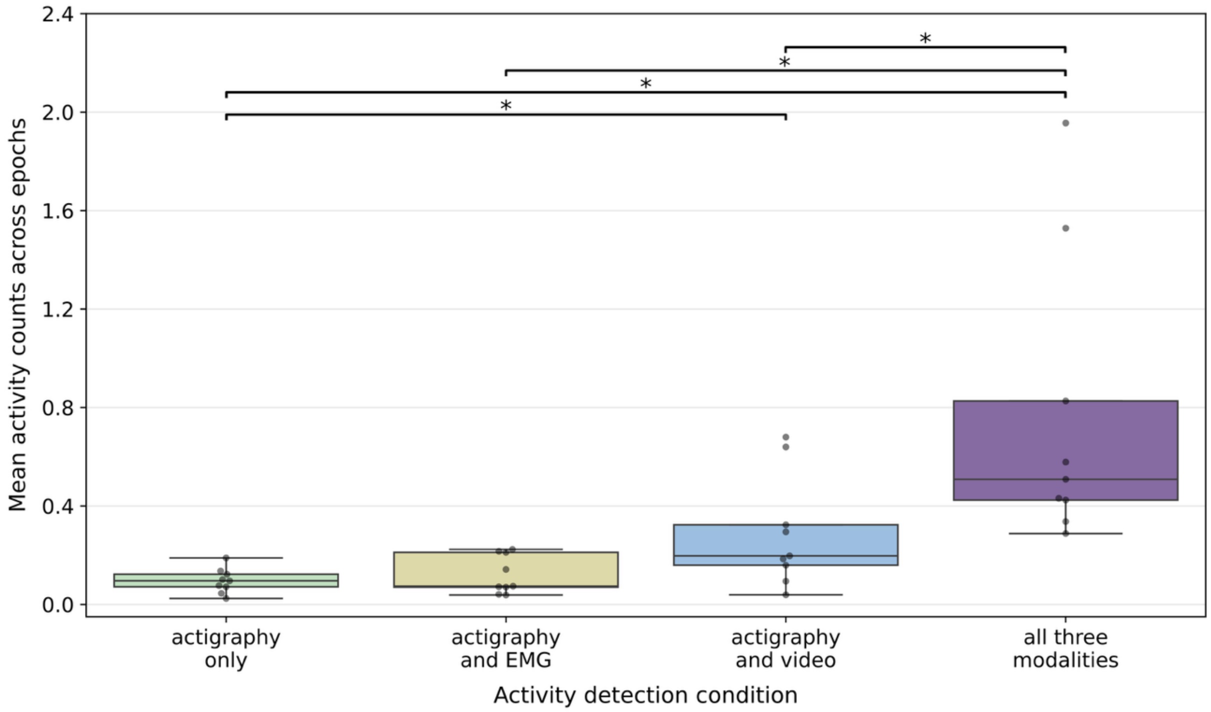

Figure S6. Full distributions of mean actigraphy activity counts during REM sleep epochs stratified by multimodal detection condition. Epochs are grouped by detection condition: detected by all three modalities (EMG, actigraphy, and video), by actigraphy and EMG, by actigraphy and video, or by actigraphy alone. Each dot represents a single Mount Sinai RBD participant, and boxplots summarize the distribution across participants. Actigraphy activity counts were highest for epochs detected by all three modalities and lowest for actigraphy-only epochs. Pairwise comparisons were performed using Wilcoxon signed-rank tests with Holm correction for multiple comparisons; asterisks denote statistically significant differences (* $p < 0.05$). This figure displays the complete (untruncated) distribution of values corresponding to Figure 6.
